# Resting-State Functional MRI Approaches to Parkinsonisms and Related Dementia

**DOI:** 10.1007/s11910-024-01365-8

**Published:** 2024-07-24

**Authors:** Noemi Piramide, Rosa De Micco, Mattia Siciliano, Marcello Silvestro, Alessandro Tessitore

**Affiliations:** 1 Department of Advanced Medical and Surgical Sciences, University of Campania “Luigi Vanvitelli”, Napoli, Italy; 2 Neuropsychology Laboratory, Department of Psychology, University of Campania “Luigi Vanvitelli”, Caserta, Italy

**Keywords:** Parkinson’s disease, Parkinsonisms, Dementia, Cognitive impairment, Resting-state, MRI

## Abstract

**Purpose of the Review:**

In this review, we attempt to summarize the most updated studies that applied resting-state functional magnetic resonance imaging (rs-fMRI) in the field of Parkinsonisms and related dementia.

**Recent Findings:**

Over the past decades, increasing interest has emerged on investigating the presence and pathophysiology of cognitive symptoms in Parkinsonisms and their possible role as predictive biomarkers of neurodegenerative brain processes. In recent years, evidence has been provided, applying mainly three methodological approaches (i.e. seed-based, network-based and graph-analysis) on rs-fMRI data, with promising results.

**Summary:**

Neural correlates of cognitive impairment and dementia have been detected in patients with Parkinsonisms along the diseases course. Interestingly, early functional connectivity signatures were proposed to track and predict future progression of neurodegenerative processes. However, longitudinal studies are still sparce and further investigations are needed to overcome this knowledge gap.

## Introduction

Cognitive impairment has been increasingly identified as a relevant condition for the majority of patients with Parkinsonism, potentially presenting from the earliest disease phases to the advanced stages [1].

Parkinsonisms are heterogeneous neurodegenerative disorders characterised by clinical parkinsonian features that may be differently associated with other motor and nonmotor symptoms [2]. The deposition of aggregated proteins into intracellular inclusion bodies is a common neuropathological denominator for these disorders, with pathological changes typically spreading into the brain over specific anatomical patterns that are characteristic for each disease.

Tau and α-synuclein are the most abundant proteins that may be found in pathological aggregates occurring typically in the presynaptic and axonal portion of neurons, but also in glial cells [1, 2].

Abnormal aggregates of α-synuclein, such as Lewy bodies (LB) and Lewy neurites have been indeed implicated in the pathophysiology of Parkinson’s disease (PD), Dementia with Lewy bodies (DLB) and Multiple systems atrophy (MSA), thereby leading to the umbrella term of synucleinopathies [1, 2].

Similarly, tauopathies are neurodegenerative disorders characterized by the deposition of abnormal tau protein in both neurons and glial cells, such as progressive supranuclear palsy (PSP), corticobasal degeneration (CBD) and frontotemporal lobar degeneration with tauopathy (FTLD-Tau) [1, 2].

However, the watershed behind this classification is blurred and mixed pathological forms have been recognized [3]. Similarly, overlapping clinical manifestations between synucleinopathies and tauopathies make the differential diagnosis challenging in the spectrum of neurodegenerative diseases [2, 3].

Cognitive impairment is common and heterogenous in PD, often extending as a continuum from subjective cognitive impairment to mild cognitive impairment (MCI) and dementia (PDD) [4].

The early pattern of PD-related cognitive dysfunction mainly involves executive and attentive/working memory domains with a slow progression over time. Memory, language and visuospatial impairments usually occur later in the disease course and have been associated to an increased risk to develop clinical overt dementia [1, 4]. This parallels with the “dual syndrome hypothesis”, that pictures the dopaminergic failure of frontostriatal connections as occurring early in the disease, leading to executive deficits with mild progression over time, whereas the involvement of the cholinergic system could determine a more “posterior” cognitive dysfunction, with higher risk to convert into dementia [1, 4].

Interestingly, along with motor (i.e. postural and gait disturbances) and demographic features (i.e. male sex, older age at onset), the presence of specific nonmotor symptoms (i.e. REM sleep behavioural disorders, neuropsychiatric symptoms, autonomic dysfunction) has been suggested to increase the risk of developing dementia in PD [1, 4].

While cognitive impairment in PD occurs usually later in the disease course with respect to motor symptoms, DLB should be diagnosed when dementia occurs before or concurrently with parkinsonism, with prominent hallucinations and visuospatial dysfunction [5].

MSA is clinically characterized by a variable combination of autonomic dysfunction, levodopa-unresponsive parkinsonism, and cerebellar signs [1–3, 6]. Cognitive disturbances were previously considered as a non-supporting feature of MSA. However, according to more recent findings and updated diagnostic criteria, cognitive symptoms have been recognized as not uncommon in this disease [7].

Among tauopathies, PSP is a rapidly progressive neurodegenerative disease with four clinical cornerstones such as ocular motor dysfunction, postural instability, akinesia and cognitive dysfunctions [8]. Cognitive deficits are present up to 58% of PSP patients at the disease onset, with an earlier presentation in the Richardson’s syndrome than Parkinsonian phenotype [1, 8]. Executive, memory and visuospatial functions are typically impaired in these patients. [6]

CBD is characterised by cortical and extrapyramidal signs. Apraxia, cortical sensory deficits and alien limb phenomena are the most common cortical signs, whereas asymmetrical parkinsonism, dystonia and myoclonus comprise the motor signs [8, 9]. The prevalence of cognitive impairment in patients with CBD is 52% at disease onset, progressively increasing up to 70% over the disease course, with language as well as visuospatial dysfunctions being the most frequent clinical syndromes [8, 9].

Neuroimaging methods have greatly improved the ability to understand the pathophysiology of Parkinsonisms, support the diagnosis of parkinsonian syndromes, and detect and monitor disease progression [10]. Among different techniques, resting-state functional magnetic resonance imaging (rs-fMRI) has been widely applied to this purpose in patients with Parkinsonisms.

Rs-fMRI is based on the spontaneous oscillation of the blood oxygen level dependent (BOLD) signals [11, 12], that derive from the processing of neuronal information at the synaptic level in specific brain areas according to the paramagnetic properties of blood [11–14]. The temporal coherence of neuronal firing patterns from different brain areas represents the so-called functional connectivity (FC) [11]. Different analytic approach may be applied to rs-fMRI data, such as seed-based FC, network-based independent component analysis and graph theory [12].

Seed-based analysis determines the FC patterns as emerging from a predefined seed or region of interest (ROI) to the whole-brain voxels or other seeds/ROIs voxels [11–14].

Independent component analysis is a data-driven method that can be applied to rs-fMRI data to isolate large-scale spatially distributed FC networks, called resting-state networks (RSNs). This method does not necessarily need a previous assumption [11–14].

Finally, graph analysis measures and techniques have been used to understand the global topological organization of brain networks. By applying this approach to rs-fMRI data, anatomic brain regions are considered to be nodes, linked by edges, which represent the FC between nodes [11–14]. Such wiring diagram of the brain is called connectome and support an efficient global integration between high-specialized segregated areas. Interestingly, it has been proposed that the connectome architecture could play a direct role in spreading misfolded proteins across the brain, also explaining the stereotypical patterns of neurodegeneration [15].

In this narrative review, we aimed at summarizing the most updated studies that applied rs-fMRI to investigate the neural correlates of cognitive impairment in patients with tau and α-synuclein-based Parkinsonisms.

### Search Strategy

Articles published in on PubMed in the last 3 years until August 2023 were systematically checked for the purpose of this review, considering only English-written articles published in peer-reviewed journals, with the use of the following words: “Parkinson’s disease”, “Lewy body dementia”, “Multiple system atrophy”, “Progressive supranuclear palsy”, “Corticobasal degeneration”, which were each cross-referenced with “resting state functional magnetic resonance imaging”.

Two independent observers (NP and RDM) evaluated the results, excluding duplicates and articles judged irrelevant by title and abstract screening. The same raters performed the quality check of selected studies and the most relevant ones for the topic were finally included in this narrative review (Tables 1, 2 and 3).

**Table 1:** Summary of the most updated MRI seed-based functional connectivity studies in patients with synucleinopathies

References	Rs-fMRI approach	Subjects	Main findings
Hou et al., Neuroradiology 2020	Seed-based analysis (DMN ROIs)	28 drug-naïve PD-MCI, 19 drug-naïve PD-CN and 28 HC	Reduced FC in the dorsomedial PFC, in temporoparietal junction, and between dorsomedial PFC and PCC in PD-MCI relative to HC
Cascone et al., Commun Biol 2021	Seed-based analysis (FPN ROIs)	37 PD-MCI, 22 PD-CN and 21 HC	Reduced topological brain-network resilience of FPN in PD-MCI patients associated with cognitive decline
Ruppert et al., Hum Brain Mapp 2021	ICA- and seed-based analysis (DMN, FPN, DAN, SMN, VN)	12 PD-MCI, 36 PD-CN and 16 HC	Increased DMN connectivity in PD-MCI compared both to PD-CN and HC. FC within DMN correlated with global cognitive, executive, attentive and visuospatial functions in PD-MCI
Wang et al., Int J Gen Med. 2021	Seed-based analysis (PCC as DMN ROI)	20 PD-MCI, 13 PD-CN and 13 HC	Decreased FC in PCC in PD-MCI patients relative to HC, correlated with worse performances information processing speed, episodic memory and general cognition
Chen et al., Front Neurosci 2022	Seed-based analysis (PCC as DMN ROI)	50 PD and 50 HC	Increased FC between the PCC and the right precuneus, left cuneus, and right angular gyrus in PD patients relative to HC supposed to be responsible for cognitive decline
Byun et al., Sleep Med 2020	Seed-based analysis (thalamus)	37 RBD and 15 HC	Increased FC between thalamus and occipital regions in RBD patients relative to HC, correlated with cognitive dysfunctions, particularly memory
Wakasugi et al., Parkinsonism Relat Disord 2021	ICA- and seed-based analysis (DMN, ECN, SMN, BGN)	50 RBD and 70 HC	Decreased FC in ECN (fronto-striatal), SMN (pre and post-central regions) and BGN in RBD patients relative to HC
Jia et al., Front Aging Neurosci 2022	Seed-based analysis (PCC as DMN ROI)	18 PD-pRBD, 28 PD-no-pRBD and 22 HC	Decreased FC in frontal regions in PD-pRBD patients, correlated with executive dysfunctions that get worse over 3 years of follow-up
Huang et al., Front Psychiatry 2020	Seed-based analysis (anterior/posterior insula)	17 PD-Dep, 17 PD-noDep and 17 HC	Decreased FC between insula and fronto-parietal regions in PD-Dep patients relative to PD-noDep patients and HC, correlated with worse global cognitive functions
Liao et al., Front Neurosci. 2020 (da controlare)	Seed-based analysis (precentral, paracentral and medial frontal gyri)	33 PD-Dep, 60 PD-noDep and 47 HC	Decreased FC between ROIs and temporo-occipital regions and as well as between precentral /medial frontral gyri and SMA in PD-Dep patients relative to PD-noDep patients and HC
Yin et al., Front Aging Neurosci 2022	Seed-based analysis	30 PD-Dep (minor), 32 PD-Dep (major), 26 PD-noDep and 30 HC	Decreased FC between temporal and cerebellar regions as well as in fronto-insular loop in PD-Dep (major) relative to PD-noDep patients, correlated with worse depressive symptoms
Chen et al., Heliyon 2023	Seed-based analysis (amygdala)	13 PD-Anx 20 PD-noAnx, and 19 HC	Decreased FC between amygdala and hippocampus, correlated with anxiety severity symptoms, as well as increased FC between the amygdala and parietal areas in PD-Anx relative to PD-noAnx patients. Reduced FC between amygdala and putamen in PD-Anx patients relative to HC
Mata-marin et al., Brain Connect 2021	ICA-based analysis (DMN, SMN, ECN and SN) and seed-based analysis (motor, associative, and limbic striatum)	17 PD-HS + , 15 PD-HS- and 17 HC	Increased FC in SN and disconnection between associative and limbic striatum with precuneus and superior parietal lobe in PD-HS +
Dujardin et al., Brain Imaging Behav. 2020	ICA- and seed-based analysis (VN and DMN)	9 PD-VH	Increased FC in VN. Increased stability in DMN correlated with VH severity
Thomas et al., Brain Commun. 2023	ICA- and seed-based analysis (LGN, medial thalamus, primary visual cortex, hippocampus and PFC as ROIs for VN)	15 PD-VH and 75 PD-no-VH	Decreased bottom-up FC from bilateral LGN to primary visual cortex; and, increased top-down FC from left PFC to primary visual cortex and medial thalamus in PD-VH relative to PD-no-VH patients
Chabran et al., Alzheimers Res Ther 2020	Seed-based analysis (key nodes as ROIs for DMN, SN, FPN and DAN)	92 DLB, 70 AD, and 22 HC	Decreased FC within SN and FPN (front-parietal ROIs), and between FPN-DAN; increased FC within DMN (medial PFC), SN (rostral PFC) and FPN (lateral PFC) in LBD patients relative to HC Decreased FC within SN and DMN, and increased FC between DMN-SN in AD patients relative to HC Decreased FC within SN (fronto-insular) and between SN-DAN, SN-FPN, SN-DMN, and increased FC within DMN (parietal/PCC) and between FPN-DAN in LBD relative to AD patients The severity of fluctuations in DLB patients positively correlated with FC between SN-DAN, and negatively correlated with FC between SN-DMN and between FPN-DMN
Schumacher et al., Brain 2021	ICA-based analysis (posterior DMN) and whole brain ROI-to-ROIs analysis	27 DLB, 26 AD and 99 HC	No differences were found between groups
Yang et al., Clin Neurophysiol 2020	Seed-based analysis (Right middle prefrontal gyrus)	32 MSA-CI, 29 MSA-CN and 33 HC	Decreased FC between right middle prefrontal gyrus and right inferior PFC, insula and precuneus in MSA-CI relative to MSA-CN patients. Decreased FC between right middle prefrontal gyrus and fronto-insular cortices in both MSA-CI and MSA-CN relative to HC. Also decreased FC with left caudate and cingulate-occipital cortices in MSA-CI relative to HC. FC alterations correlated with global cognitive performances in MSA-CI

Abbreviations: *Rs-fMRI*, resting-state functional magnetic resonance imaging; *AD*, Alzheimer’s disease; *CI*, Cognitive impaired; *CN*, cognitive normal; *DAN*, dorsal attentive network; *DLB*, Dementia wit Lewy Body; *DMN*, default mode network; *ECN*, Executive control network; *FC*, functional connectivity; *FPN*, frontoparietal network; *HC*, healthy controls; *HS*, hypersexual; *ICA*, Independent Component Analysis; *LGN*, lateral geniculate nucleus; *MCI*, Mild Cognitive Impairment; *MSA*, multiple sistem atrophy; *PD*, Parkinson’s disease; *PFC*, prefrontal cortex; *SMN*, sensorimotor network; *SN*, salience network; *inSN*, medial SN; *laSN*, lateral SN; *pRBD*, probable RBD; *RBD*, REM sleep behavior disorder; *ROIs*, Regions of interest; *VH*: visual hallucinations; *VN*, visual network

**Table 2:** Summary of the most updated MRI ICA-based functional connectivity studies in patients with synucleinopathies

References	Rs-fMRI approach	Subjects	Main findings
Schindlbeck et al., Cereb Cortex. 2021	ICA-based analysis (DMN)	Cohort 1: 23 cognitive impaired PD 15 HC Cohort 2: 22 cognitive impaired PD 19 HC	Reduced FC in the ventral DMN, mainly the PCC and precuneus, in cognitively impaired PD patients. Alterations involving the lateral prefrontal and posterior parietal regions, medial temporal cortex, and pons correlated with cognitive dysfunction
Hou et al., BMC Neurol. 2021	ICA-based analysis (DMN, DAN, FPN, SMN, VAN, VN and limbic network)	28 drug-naïve PD-MCI, 19 drug-naïve PD-CN and 28 HC	Reduced FC within DMN (right precuneus), SMN (left paracentral gyri and SMA), and VN (bilateral calcarine and lingual) in PD-MCI relative to HC. Reduced FC between SMN-limbic network and VN-VAN in PD-MCI and PC-CN relative to HC Lower FC within SMN in all PD patients correlated with disease severity Lower FC in DMN and VN correlated respectively with worse memory and visuospatial performances in PD-MCI
Ruppert et al., Hum Brain Mapp 2021	ICA- and seed-based analysis (DMN, FPN, DAN, SMN, VN)	12 PD-MCI, 36 PD-CN and 16 HC	Increased DMN connectivity in PD-MCI compared both to PD-CN and HC. FC within DMN correlated with global cognitive, executive, attentive and visuospatial functions in PD-MCI
Wakasugi et al., Parkinsonism Relat Disord 2021	ICA- and seed-based analysis (DMN, ECN, SMN, BGN)	50 RBD and 70 HC	Decreased FC in ECN (fronto-striatal), SMN (pre and post-central regions) and BGN in RBD patients relative to HC
De Micco et al., J Neural Transm (Vienna) 2023	ICA-based analysis (DMN, FPN, ECN, SN)	25 PD-pRBD, 32 PD PD-no-pRBD and 23 HC	Decreased FC within the FPN and increased FC within the DMN, ECN and SN in PD-pRBD patients PD-no-pRBD, while decreased FC in the DMN, FPN, ECN and SN in PD-pRBD patients relative to HC
Lin et al., Neuroimage Clin 2020	ICA-based analysis (DMN, subcortical network, AUD, SMN, VN, ECN and cerebellar newtwork)	59 PD-Dep, 97 PD-noDep and 45 HC	Decreased FC within insula and between insula and PCC/precuneus, and increase FC within PCC, between PCC and hippocampus + amygdala, and between fronto-parietal areas in PD-Dep relative to PD-noDep patients, correlated with depressive symptoms
Xu et al., Front Neurosci 2022	ICA-based analysis (DMN, AUD, DAN, FPN, SMN, VAN, VS and SN)	21 de novo PD-Dep, 34 de novo PD-noDep and 43 HC	Decreased FC within VAN (middle temporal cortex) in de novo PD-Dep patients and PD-noDep patients relative to HC, correlated negatively with depressive symptoms severity Decreased FC between AUD-DMN/VAN in PD-Dep relative to PD-noDep patients, decreased FC between AUD-DMN/VAN/VIS, DAN-DMN and between VAN-VIS as well as increased FC between FPN-VAN in PD-Dep patients relative to HC
Liu et al., Frontiers neurol 2022	ICA-based analysis (aDMN, spDMN, ip DMN, FPN, precuneus network, BGN and inSN)	42 PD with moderate-severe Dep, 29 PD with mild Dep and 35 PD-noDep	Increased FC in the spDMN, but it was decreased in the LFPN and inSN networks in all groups relative to HC. Increased FC in the spDMN and decreased in the LFPN and inSN networks in the in PD with mild and moderate-severe Dep relative to PD-noDep patients. Decreased FC inSN in PD with moderate-severe Dep relative to PD with mild Dep patients
De Micco et al., Mov Disord 2021	ICA-based analysis (DMN, FPN, SMN, ECN and SN)	25 drug naïve PD-anx, 25 drug naïve PD-noAnx and 20 HC	Decreased FC within DMN, FPN (fronto-temporal), SN (ACC) and SMN, as well as increased FPN (frontal), SN (insular) and ECN (cingulum) in PD-anx relative to PD-noAnx patients, correlated with anxiety severity symptoms. Decreased FC within DMN, FPN,SN, SMN and ECN in PD-anx and PD-noAnx relative to HC Decreased FC between SN-ECN, SN-SMN and SN-FPN in PD-anx relative to PD-noAnx patients
Mata-marin et al., Brain Conn 2021	ICA-based analysis (DMN, SMN, ECN and SN) and seed-based analysis (motor, associative, and limbic striatum)	17 PD-HS + , 15 PD-HS- and 17 HC	Increased FC in SN and disconnection between associative and limbic striatum with precuneus and superior parietal lobe in PD-HS +
Dujardin et al., Brain Imaging Behav. 2020	ICA- and seed-based analysis (VN and DMN)	9 PD-VH	Increased FC in VN. Increased stability in DMN correlated with VH severity
Thomas et al., Brain Commun. 2023	ICA- and seed-based analysis (LGN, medial thalamus, primary visual cortex, hippocampus and PFC as ROIs for VN)	15 PD-VH and 75 PD-no-VH	Decreased bottom-up FC from bilateral LGN to primary visual cortex; and, increased top-down FC from left PFC to primary visual cortex and medial thalamus in PD-VH relative to PD-no-VH patients
Schumacher et al., J Neurol 2021	ICA-based analysis (DMN, VN, BGN, AN, motor, temporal, frontal and cerebellar networks	31 DLB-MCI, 28 AD-MCI and 24 HC	No differences were found between MCI-AD and MCI-DLB patients
Schumacher et al., Brain 2021	ICA-based analysis (posterior DMN) and whole brain ROI-to-ROIs analysis	27 DLB, 26 AD and 99 HC	No differences were found between groups

Abbreviations: *Rs-fMRI*, resting-state functional magnetic resonance imaging; *AD*, Alzheimer’s disease; *AN*, attentive network; *AUD*, auditory network; *C*: cerebellar; *CN*, cognitive normal; Dep, depression; *DAN*, dorsal attentive network; *DLB*, Dementia wit Lewy Body; *DMN*, default mode network; *aDMN*, anterior *DMN*; ip DMN, posterior superior DMN; *spDMN*, superior posterior DMN; *ECN*, Executive control network; *FC*, functional connectivity; *FPN*, frontoparietal network; *HC*, healthy controls; *HS*, hypersexual; *ICA*, Independent Component Analysis; *LGN*, lateral geniculate nucleus; *MCI*, Mild Cognitive Impairment; *PD*, Parkinson’s disease; *PFC*, prefrontal cortex; *SMN*, sensorimotor network; *SN*, salience network; *inSN*, medial SN; *pRBD*, probable RBD; *RBD*, REM sleep behavior disorder; *ROIs*, Regions of interest; *VAN*, ventral attentive network; *VH*: visual hallucinations; *VN*, visual network

**Table 3:** Summary of the most updated MRI graph-analysis functional connectivity studies in patients with synucleinopathies

References	Rs-fMRI approach	Subjects	Main findings
Hou et al., J Neurol sci 2020	Graph-analysis	22 drug-naïve PD-MCI, 19 drug-naïve PD-CN and 28 HC	Decreased clustering coefficient, local efficient and path length, and increased global coefficient in PD-MCI and PD-CN relative to HC. Decreased nodal centralities in SMN, DMN and the ventral aPFC, and increased nodal centralities in nodes of the cingulo-opercular network, occipital network, and the ventral lPFC in PD-MCI patients relative to HC. Increased nodal centrality in the cingulo-opercular network negatively correlated with cognitive scores
Chen et al., Front Neurosci 2020	Graph-analysis	45 early PD-MCI, 22 early PD-CN and 18 HC	Decreased clustering coefficient and small-world index, increased characteristic path length, increased nodal centrality in DMN, ECN, VN and decreased nodal centrality in SMN in PD-MCI relative to PD-CN and HC
Suo et al., Cerebral cortex 2022	Graph-analysis	24 PD-MCI, 17 early PD-CN and 24 HC	At the global level, decreased clustering coefficient, global efficiency and local efficiency, and increased path length in PD-MCI and PD-CN relative to HC; path length and global efficiency were correlated with language alterations in PD-MCI. At the regional level, decreased nodal metrics in sensorimotor regions in PD-MCI and PD-CN relative to HC. Lower intramodular connectivity in DMN, subcortical-cerebellum loop, and lower intermodular connectivity between DMN and FPN in PD-MCI relative to DP-CN correlated with cognitive global scores
Campabadal et al., Neuroimage Clin 2020	Graph-analysis	20 RBD and 25 HC	Decreased FC strength in temporo-parietal areas, correlated with mental processes slowness, and lower nodal centrality in parietal lobe in RBD patients relative to HC
Li et al., Front. Neurol 2020	Graph-analysis	30 PD-pRBD, 62 PD-no-pRBD and 20 HC	Decreased betweenness centrality in the right dorsolateral superior frontal gyrus as well as increased nodal efficiency in the bilateral thalamus correlated with RBD severity symptoms, and increased betweenness centrality in the insula in PD-pRBD relative to PD-no-pRBD patients. Increased nodal efficiency, degree centrality, and between centrality in fronto-limbic, subcortical and pareital areas in PD-pRBD relative to HC. Decreased nodal clustering coefficient in frontal lobe, nodal efficiency in parietal/occipital lobe, nodal centrality in occipital lobe in PD-pRBD relative to HC
Oltra et al., Scientific Reports 2021	Graph-analysis	27 PD-pRBD, 32 PD-no-pRBD and 30 HC	Decreased FC strength and increased path length in posterior regions in PD-pRBD patients relative both to PD-no-RBD patients and HC, correlated with worse visuoperceptual, processing speed and verbal memory functions
Naval-Potro et al., Park related disord 2020	Graph-analysis	16 PD-ICD, 20 PD-noICD and 17 HC	Increased local efficiency in SN in PD-ICD relative to PD-noICD patients and HC
Zheng et al., Aging 2020	Graph-analysis	24 MSA-C and 20 HC	Decreased local efficiency and weighted degree in cerebellum, and weighted degree in vermis 6; increased betweenness centrality in dorsolateral PFC and crus 9 of cerebellu in MSA patients relative to HC. Decreased weighted degree in vermis 6 and increased betweenness centrality in dorsolateral PFC and crus 9 of cerebellum correlated with MSA severity
Ge et al., Front Aging Neurosci. 2022	Graph-analysis	29 MSA-C and 27 HC	Decreased FC between cerebellum lobues and fronto-parietal areas, and increased FC between intra-cerebellar regions, and between cerebellum and fonto-temporal areas /ACC in MSA-C patients
Chen et al., Hum Brain Mapp. 2023	Graph-analysis	76 MSA-P, 53 PD and 88 HC	Decreased local efficiency in MSA-P relative to HC, and decreased cluster coefficient in both groups relative To HC. Decreased nodal centralities in fronto-temporal regions in both MSA-P and PD patients relative to HC, negatively correlated with disease severity in the second group Decreased FC intercerebellar and cerebellar‐DMN in MSA-P relative to PD patients; decreased FC in the DMN and BGN in PD relative to MSA-P patients

Abbreviations: *Rs-fMRI*, resting-state functional magnetic resonance imaging; *C*: cerebellar; *CN*, cognitive normal; *DMN*, default mode network; *ECN*, Executive control network; *FC*, functional connectivity; *FPN*, frontoparietal network; *HC*, healthy controls; *MCI*, Mild Cognitive Impairment; *MSA*, multiple sistem atrophy; *MSA-C*, cerebellar MSA; *MSA-P*, parkinsonian MSA; *PD*, Parkinson’s disease; *PFC*, prefrontal cortex; *aPFC*, anterior prefrontal cortex; *lPFC*, lateral prefrontal cortex; *SMN*, sensorimotor network; *SN*, salience network; *pRBD*, probable RBD; *RBD*, REM sleep behavior disorder; *VAN*, ventral attentive network; *VN*, visual network

### Functional MRI Studies in Patients with Synucleinopathies (Tables 1, 2 and 3)

#### PD and PDD

Heterogeneous patterns of rs-fMRI FC alterations were found to be associated to PD-related cognitive impairment. This may be potentially due to the inclusion of small samples, patients at different disease stages and/or in different medication states, and to the application of various fMRI approaches [16–18]. In the last three years, several studies have been performed in patients with PD-MCI, sometimes leading to conflicting results [17, 19]. A few studies have been performed in patients with PDD.

The most reported large-scale RSN associated with cognition in PD is the default-mode network (DMN) [17, 19], mainly involved in self-centered mental imagery, mind-wandering and episodic memory retrieval. Several studies reported the presence of decreased FC within the DMN as associated with cognitive processing deterioration in PD [17–19]. Recent findings have confirmed these results showing that decreased FC in DMN is correlated with episodic memory/working memory, executive/attentive or altered information processing speed, visuospatial dysfunctions and worse general cognition [20–23]. A similar pattern of DMN involvement, particularly its posterior hubs, has been found also in early and/or drug-naive PD-MCI patients and in the absence of grey matter atrophy [20–23], suggesting that FC rearrangements within this network may reflect the presence of pathological processes (i.e. as misfolded protein accumulation) that not directly lead to neuronal death but may induce synaptic activity disturbances, which have been correlated with rs-fMRI signal strength [13]. These correlates may be detected early in the disease and may be potentially tested as biomarkers to monitor disease progression and treatment-response.

On the other hand, also increased FC within DMN has been reported in PD-MCI patients and in cognitive normal patients (PD-CN) in advanced stages [24, 25].

Even though decreased FC may be straightforward associated to loss of neural function, it is important to note that “hyper-connectivity” has also pathologic implications. Indeed, increased FC may be compensatory, representing a network response to the local neuronal injury that allows for the maintenance of the same global performance in non-demented PD patients to delay the development of clinical overt dementia. Moreover, what we see as “hyper-connected” may be underlined by the loss of networks dynamic properties to shift between different states (i.e. from a “hyper- “ to a “hypo-connected” state), which has been also related to the presence of cognitive impairment in PD [26].

This is in line with the finding of decreasing FC within the DMN over 3 years of follow-up in PD patients, along with progressive cognitive decline [27].

Together with DMN, other relevant RSNs such as the frontoparietal (FPN), dorsal and ventral attention (DAN/VAN), salience (SN), executive-control (ECN) and visual (VN) networks, have been studied to investigate the potential association with cognitive impairment in PD.

The FPN is mainly involved in decision making and executive functioning. Decreased FC within this network has been consistently found in PD-MCI patients, also associated with progressive visuospatial, memory and attention dysfunctions [28–33].

The DAN plays a key-role in the top-down processes to control voluntary movements while the VAN is involved in the bottom-up detection of relevant stimuli [19]. Decreased FC has been revealed within both DAN and VAN as associated with impaired attention and executive functioning, suggesting the presence of altered high cognitive control in PD-MCI [29, 32, 34, 35].

Two recent studies have focused the amnestic (aMCI) and non-amnestic (naMCI) subtypes of PD-related MCI, showing divergent findings. Indeed, Chung and colleagues [29] performed a multimodal MRI study and found increased FC within the SN in PD-aMCI compared to PD-naMCI patients. Interestingly, in a longitudinal sub-cohort of PD, patients with PD-aMCI patients exhibited a higher risk of conversion to dementia compared to PD-naMCI. Conversely, Kawabata et al. revealed the presences of DMN changes characterising PD patients with aMCI from healthy controls (HC), while VN and cerebellar-brainstem network was found to be specifically altered in naMCI [36]. These conflicting results may stem from the inclusion of different PD samples along with the application of heterogenous diagnostic criteria and methodological rs-fMRI approaches. Nonetheless, these findings support previous evidence of different cognitive syndromes in PD patients [1, 4] that may not include an Alzheimer’s disease (AD)-like phenotype [37], determining a slower but progressive multidomain deterioration of the brain efficiency and functioning that might eventually lead to dementia [38].

Beyond FC alterations within each network, the interaction between different RSNs has been found to be associated with cognitive impairment in PD patients [17, 20, 28, 34, 39, 40]. Overall, a functional coupling/decoupling patterns have been reported between the RSNs, with specific directions that seem critical to generate and maintain an efficient behavioral and cognitive performance, with several evidence in PD patients [17, 20, 28, 34, 39–42].

Graph-analysis MRI studies also provided intriguing findings to support the pathophysiology of PD-related cognitive dysfunction. Overall, altered connectomic metrics within the DMN, FPN and VN have been found to be associated with cognitive impairment in PD patients, suggesting the presence of both local and global information processing changes of the brain architecture [24, 30, 43, 44]. Indeed, a progressive shift from a global integrated to a small-world highly segregated organisation has been proposed to determine the development of MCI in PD. This would allow PD-MCI patients to confine affected nodes which are direct targets of early degenerative processes, while potentiating still-functioning networks to maintain an efficient brain functioning at a clinical level.

Despite the heterogeneity in rs-fMRI methodology, compelling evidence suggests the influence of the dopaminergic replacement therapy (DRT) on these neural patterns [45, 46]. A recent study [40] in early PD patients showed that along with the normalization of FC within motor networks, acute levodopa administration also enhances FC metrics in nonmotor areas (i.e. frontal and limbic regions). Further studies are needed to clarify whether this effect may be detrimental for brain functioning, likely leading to the development of cognitive and behavioural disturbances over time.

The presence of specific nonmotor symptoms, such as REM sleep behavioural disorders (RBD), depression, anxiety, impulsive compulsive disorders (ICD) and visual hallucinations (VHs), have been associated with an increased risk of dementia in PD patients. Interestingly, rs-fMRI studies allowed to highlight specific changes over cognitive-related brain regions and networks in PD patients with these symptoms, even in the absence of clinical overt cognitive impairment.

RBD is a parasomnia characterized by loss of atonia during REM sleep [4, 47]. RBD may present as a prodromal symptom of PD, occurring even 10 years before the onset of motor features [4, 47], and in the absence of overt signs of neurodegeneration (idiopathic RBD). Epidemiological studies recognized RBD as a risk factor for future development of cognitive impairment in PD patients [48, 49]. Previous studies have reported decreased FC within striato-frontal and temporo-parietal areas [50–52] as well as increased FC between basal ganglia and occipital cortex in patients with idiopathic RBD [53]. Widespread FC disruption and potential maladaptive compensatory changes involving mainly the dorsolateral prefrontal cortex (PFC) and posterior cortical regions have been found in cognitively unimpaired PD patients with RBD, correlated with attention and memory cognitive outcomes [52, 54–56]. The presence of such FC rearrangements may be tested as a potential future biomarker of an early vulnerability to develop dementia [54], as often seen in PD patients with RBD.

Neuropsychiatric symptoms including depression and anxiety have a great impact on PD patients’ quality of life, motor disability and caregiver burden. Their association with cognitive impairment in PD patients has been already reported, also suggesting a predictive role for the future development of dementia [57, 58].

In PD patients with depressive symptoms, previous imaging studies have demonstrated the presence of hypoconnectivity in fronto-temporo-parietal, insular and cerebellar areas as well as hyperconnectivity in limbic regions, that correlated with symptoms severity and cognitive deficits, particularly executive/attentive, even in the earliest stages [59–66]. Overall, these changes have been proposed to be associated to the failure of frontal-limbic modulation of cognitive control, affective processing and emotional regulation [59–66]. Moreover, a disrupted top-down cognitive control over cortico-limbic networks has been hypothesized to be involved in the onset/maintenance of depression in PD patients [67, 68].

Epidemiological studies have found a higher prevalence of anxiety symptoms in PD patients with cognitive impairment [57, 58]. Both decreased and increased striato-limbic and posterior FC was found in PD patients with anxiety correlated with severity of anxiety symptoms [58, 63, 69, 70]. Taken together, these findings support the hypothesis that decreased top-down emotion regulation, limbic-prefrontal disconnection and abnormal cognitive control may represent potential pathophysiological mechanisms underlying neuropsychiatric symptoms in PD that may potentially lead to the development of cognitive impairment over time.

Clinical and neuroimaging evidence support the presence of a link between cognition and ICD in PD patients [17]. Increased FC within striato-limbic and posterior regions has been associated with impulsivity in PD patients with ICD [71–73], potentially linked to increased perception of reward stimuli and internally-oriented ruminating compulsion. Similarly, decreased FC within the fronto-striatal network has been associated with stronger impulsivity and weaker behavioural control on decision making, leading to risky or inappropriate actions [71–74]. These findings support the crucial role of cognitive functioning to maintain efficient risk/benefit evaluation and decision-making, which may explain the presence of FC changes over cognitive-related regions.

Finally, VHs are common nonmotor symptoms in PD, regarding up to 40% of patients [75, 76]. Their presence is commonly linked to a higher risk of cognitive decline and dementia over time [77]. In PD patients with VHs, previous rs-fMRI studies have demonstrated the presence of decreased FC within temporo-occipital areas and thalamic-visual network as well as increased FC in VN and between thalamus and frontal areas. Overall, these data suggest that altered integration of high order visual and cognitive processing may underlie the development of VHs in these patients [78–80].

#### DLB

DLB is the primary diagnosis of approximately 5% of patients with dementia [81]. Clinically, patients with DLB present with dementia which may be variously associated with hallucinations, cognitive fluctuations, parkinsonism, and RBD.

In the last 3 years, only a few studies have been published that investigated the potential FC changes associated to DLB, also reporting controversial results with respect to previous evidence [81, 82].

Two studies reported a similar pattern of FC alterations within the posterior DMN in DLB and AD patients compared to HC, even in the absence of clinical overt dementia [83, 84]. When compared to HC, PDD and AD patients, DLB patients showed disrupted FC within the DMN (mainly the anterior portion), FPN, SN, SMN, VAN as well as within striato-frontal, striato-temporal, striato-occipital, temporo-occipital and parieto-occipital areas and cerebellar regions [81, 82, 85–91]. Moreover, the presence of functional decoupling within the most reported neurocognitive networks has also been reported [81, 82, 85–91]. Overall, a mismatch between the bottom-up inputs from the visual network and the top-down processing of visual stimuli from the prefrontal areas has been proposed to underlie the development of DLB core symptoms [81, 82]. On the other hand, other studies reported increased FC within the DMN (mainly the posterior portion), the VAN as well as between basal ganglia and posterior cortices, fronto-parietal and visuoperceptual areas in DLB patients relative to HC and AD [81, 86, 90], that have been hypothesized to exert a potential compensatory role.

#### MSA

MSA is a rare synucleinopathy characterized primarily by the presence of autonomic dysfunction associated with parkinsonian (i.e. MSA parkinsonian variant, MSA-P) or/and cerebellar (i.e. MSA, cerebellar variant, MSA-C) symptoms [6, 92]. Moreover, approximately 30% of MSA patients presents MCI, with executive/attentive, visuospatial and verbal functions being the most impaired cognitive domains [6]. Most MRI studies in MSA patients have been focused on brain structural alterations, while a few reports have explored functional neural correlates in these patients.

Rs-fMRI studies have compared MSA patients with PD patients and HC, considering the two variants together and also separately, leading to controversial results. Many studies demonstrated the presence of disrupted FC within the DMN, SMN, VN and cerebellar regions, and between the cerebellum and the neurocognitive networks in MSA patients, particularly in MSA-C, relative to HC and PD patients [93–99]. FC changes within the cerebellum-striato-cortical network have been also associated with the presence of cognitive dysfunction in these patients. Interestingly, in patients with MSA, FC changes within the cerebello-prefrontal network have been associated with verbal fluency and memory deficits whereas disconnection within the cerebello-limbic/temporal loop has been involved in language and visuospatial impairment [96, 100].

Functional rearrangements within cognitive-related brain areas have been found in both MSA clinical phenotypes. Indeed, an increased FC between the dentate nucleus and posterior cingulate cortex has been demonstrated in MSA-P relative to PD patients and HC, while an increased FC between cerebellar and temporo-parietal regions and within the ponto-cerebellar network was found in MSA-C patients relative to HC [93, 95, 99], with a potential compensatory role.

Finally, increased FC between cerebellum and frontal areas/anterior cingulate cortex was also reported in MSA patients and supposed to be related to neuropsychiatric symptoms such as anxiety and/or depression [95].

### Functional MRI Studies in Patients with Tauopathies (Table 4)

**Table 4:** Summary of the most updated MRI functional connectivity studies in patients with tauopathies

References	Rs-fMRI approach	Subjects	Main findings
Aghakhanyan et al., EJNMMI 2022	Network-based statistics	14 PSP-RS, 10 PSP-noRS, 13 no-AD MCI	Decreased FC in DMN, SN, motor and FPN, and between cerebellum and subcortical and limbic structures, as well as increased FC between secondary visual cortex and inferior temporal gyrus, and between the cerebellum and cortical associative nodes in PSP-RS relative to non-AD MCI patients
Whiteside et al., Human Brain Mapp 2023	ICA-based analysis (DMN, SMN, FPN, DAN and VN)	97 PSP, 71 CBS, and 81 HC	Decreased FC between VN and SMN in PSP patients relative to HC Decreased FC between DMN, DAN, and FPN in CBS patients relative to HC FC in both groups correlated with more rapid decline in severity
Ballarini et al., Neuroimage Clin 2020	Seed-based analysis (superior temporal gyrus, inferior frontal gyrus, anterior PFC, bilateral caudate and middle frontal gyri)	19 CBS and 19 HC	Decreased FC in temporo-parietal and insular regions, cingular and posterior cortices as well as increased FC in the frontal-basal ganglia loop in CBS relative to HC
Piervincenzi et al., Biomedicines 2023	Seed-based analysis (SMA, primary motor area, putamen, globus pallidus and thalamus)	12 FTD-Park + , 18 FTD-Park- and 30 HC	Decreased FC between SMA and putamen in FTD-Park + relative to FTD-Park- patients and HC

Abbreviations: *Rs-fMRI*, resting-state functional magnetic resonance imaging, *AD*, Alzheimer’s disease; CBS, corticobasal syndrome; *DAN*, dorsal attentive network; *DMN*, default mode network; *FC*, functional connectivity; *FPN*, frontoparietal network; *FTD*, frontotemporal dementia; *HC*, healthy controls; *ICA*, Independent Component Analysis; *Park*, parkinsonian variant; *PSP*, Progressive supranuclear palsy; *PSP-RS*, PSP Richardson syndrome variant; *SN*, salience network; *ROIs*, Regions of interest

#### PSP

Among the tauopathies, PSP is a heterogeneous neurodegenerative disorder [8], with several phenotypic variants. PSP-Richardson’s syndrome (PSP-RS) and PSP parkinsonian variant (PSP-P) are the most frequent [8]. Beyond postural instability and oculomotor dysfunction, the great majority of PSP patients present also with early cognitive impairment. Frontal executive and verbal fluency dysfunction are the most characteristic and early deficits in PSP patients [8]. However, memory, naming, visuospatial and social cognition deficits may also develop in these patients over the disease course [8]. Growing and consistent structural MRI findings have increased the interest on neuroimaging correlates that may support clinical and pathological features in PSP [8, 101]. However, very little is known about rs-fMRI neural patterns that may be associated to the clinical spectrum of cognitive deficits in PSP.

Most studies demonstrated the presence of decreased FC within the most reported rs-fMRI networks, particularly in prefrontal areas and basal ganglia, that are associated with worse cognitive performances. Decreased FC within the midbrain has also been found and related to worse executive functions and vertical gaze impairment. Finally, rs-fMRI alterations within the thalamus and cerebellum have been also reported in PSP patients [102–109]. Contrarily, some studies demonstrated the presence of increased FC within the DMN and the thalamo-cerebello-midbrain pathway, that correlated with worse cognitive status. Thus, this pattern could potentially underlie the presence of maladaptive FC between these areas, that eventually exert a detrimental effect on cognitive processing.

The presence of FC changes within the neurocognitive networks was also recently investigated in studies comparing PSP-RS and PSP-P patients, with PSP-RS showing more diffuse alterations within the DMN, SN, FPN networks as well as motor, limbic, cerebellar, occipito-temporal and thalamic areas potentially linked to increased neuropathological changes [108, 110].

#### CBD/FTD

CBD is a rare and progressive tauopathy with different clinical presentations [9, 111]. The most common presentation of CBD is the corticobasal syndrome (CBS). Together with parkinsonian symptoms, CBS patients present with altered high cortical functioning and behavioral changes [9]. To date, rs-fMRI evidence in these patients is scarce.

Decreased FC between thalamus and fronto-striatal and cerebellar regions as well as between lateral VN and auditory networks were found in CBS patients relative to HC [106, 109]. Decreased FC within the cortico-subcortical-thalamic network may reflect the presence of specific neuropathological changes in these regions, that may in turn explain the presence of parkinsonian signs/symptoms. Similarly, the functional disconnection between auditory and visual networks may be related to altered high order multisensory inputs integration that may be related to the presence of cortical signs/symptoms in CBS patients [106, 109, 111]. On the other hand, an increased FC has been demonstrated in CBS patients relative to HC within the DMN, SMN, ECN, cerebellar, fronto-cerebellar and insular networks, and have been supposed to be linked to altered motor planning-preparation and executive/emotional control on movements [106, 109, 111, 112].

To the best of our knowledge, only one study has explored rs-fMRI changes in patients with Fronto-temporal dementia and parkinsonism (FTD-P), demonstrating the presence of decreased FC between the striatum and the supplementary motor area compared to HC, potentially responsible for failure in cognitive and motor processing in these patients [113].

## Conclusion

This review resumes the most updated findings of rs-fMRI studies in patients with Parkinsonisms and related dementia.

Overall, in patients with Parkinsonisms and cognitive impairment, both synucleinopathies and tauopathies, aberrant increased and/or decreased FC alterations within the most reported neurocognitive networks have been found, further supporting a potential role for rs-fMRI as a surrogate biomarker of cognitive outcome.

Interestingly, FC changes within cognitive-related areas have been also found in PD patients with specific nonmotor symptoms which are known to be associated with high risk of dementia, even in the absence of cognitive impairment. This could potentially support the identification of early neural correlates that may be used to predict future conversion to dementia in PD patients.

While consistent results have been collected in patients with PD, a more intricate scenario emerged in other Parkinsonisms, with some studies showing also conflicting findings. More complex neuropathological patterns, also including the coexistence of different types of protein aggregates, as well as heterogenous clinical presentations compared to PD, may potentially explain this issue, still limiting the use of rs-fMRI as a reliable tool to help clinicians in the differential diagnosis.
